# Low-Dimension Nanomaterial-Based Sensing Matrices for Antibiotics Detection: A Mini Review

**DOI:** 10.3389/fchem.2020.00551

**Published:** 2020-07-24

**Authors:** Yucan Dong, Fengting Li, Ying Wang

**Affiliations:** ^1^State Key Laboratory of Pollution Control and Resources Reuse, College of Environmental Science and Engineering, Tongji University, Shanghai, China; ^2^Shanghai Institute of Pollution Control and Ecological Security, Shanghai, China

**Keywords:** nanomaterials, antibiotics detection, low-dimension, fluorescent, electrochemical

## Abstract

Antibiotics, a kind of secondary metabolite with antipathogen effects as well as other properties, are produced by microorganisms (including bacterium, fungi, and actinomyces) or higher animals and plants during their lives. Furthermore, as a chemical, an antibiotic can disturb the developmental functions of other living cells. Moreover, it is impossible to avoid its pervasion into all kinds of environmental media via all kinds of methods, and it thus correspondingly becomes a trigger for environmental risks. As described above, antibiotics are presently deemed as a new type of pollution, with their content in media (for example, water, or food) as the focus. Due to their special qualities, nanomaterials, the most promising sensing material, can be adopted to produce sensors with extraordinary detection performance and good stability that can be applied to detection in complicated materials. For low-dimensional (LD) nanomaterials, the quantum size effect, and dielectric confinement effect are particularly strong. Therefore, they are most commonly applied in the detection of antibiotics. This article focuses on the influence of LD nanomaterials on antibiotics detection, summarizes the application of LD nanomaterials in antibiotics detection and the theorem of sensors in all kinds of antibiotics detection, illustrates the approaches to optimizing the sensitivity of sensors, such as mixture and modification, and also discusses the trend of the application of LD nanomaterials in antibiotics detection.

## Introduction

Since their discovery in 1929, antibiotics have been extensively adopted for many decades. At present, there is abundant research on the content of antibiotics in water bodies and food as well as the transportation, transformation, and degeneration patterns of antibiotics in nature. Although most antibiotics do not have long half-lives, they can be regarded as chronic organic pollutants due to their long-term and lasting use. Antibiotics are harmful to the human body as they can alter the microbial community inside it, disturb the human metabolism, and produce antibiotic-resistant genes and antibiotic-resistant bacteria in the environment (Kuemmerer, 2009; Ben et al., 2019). The concentration of antibiotics in wastewater ranges from a few ng to tens of thousands of μ*g*. Nonetheless, antibiotics are rarely detected in other environmental media such as air, and the residue in soil mainly comes from irrigation (Mohammad-Razdari et al., 2019a).

At present, detection of the amount of antibiotics has already achieved a relatively low limit of quantitation. For example, by combining solid-phase extraction with liquid chromatography, the quantitation limit can be taken to an order of magnitude lower than 10^−12^ M. However, the instrument mentioned above is bulky and difficult to operate (Netea et al., 2019). In terms of innovation, nanomaterials have four distinctive properties, namely their surface effect, quantum size effect, quantum tunnel effect, and dielectric confinement effect. LD materials have a high specific surface area, with quick electron conduction. Moreover, the high abundance of surface defects helps them to emit high-intensity fluorescence, which is an excellent characteristic for both the detector of the sensor and the signal sensing module (Coleman et al., 2006). Sensors produced with this kind of material are easy to operate, quick in detection, and highly sensitive, making them the primary direction of development in this field at present. This article summarizes work on the application of 0-, 1-, and 2-dimensional nanomaterials in antibiotics detection, respectively, explores the application mechanism of nanomaterials, and puts forward the development trend in this field.

## The Role of Nanomaterials in Antibiotics Detection

**Figure 1:**
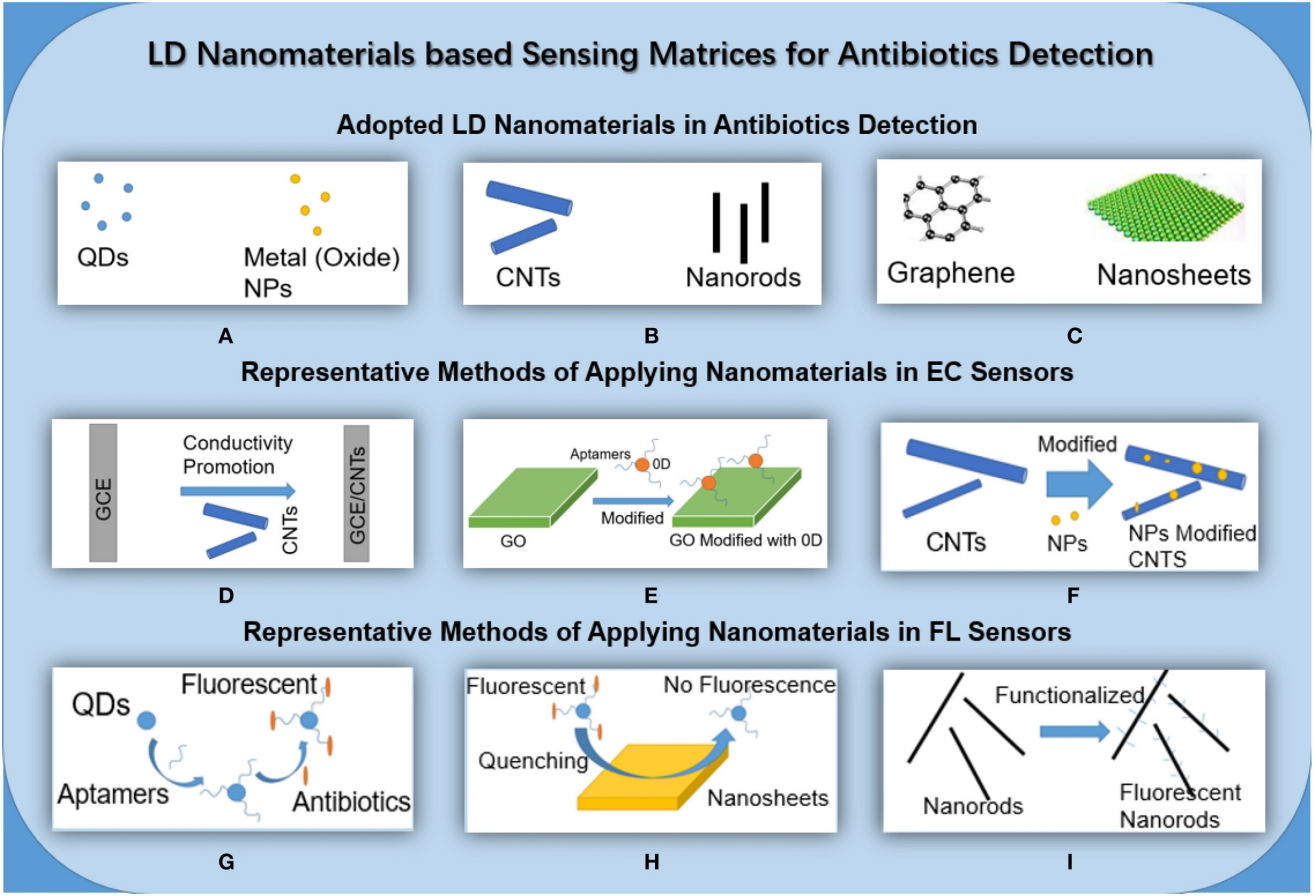
Schematic diagram of LD nanomaterials for EC and FL. **(A)**. 0D nanomaterials commonly used in the construction of sensors for antibiotic detection. **(B)** 1D nanomaterials commonly used in the construction of sensors for antibiotic detection. **(C)** 2D nanomaterials commonly used in the construction of sensors for antibiotic detection. **(D)** A common strategy for applying nanomaterials in EC sensors: modify the electrode with CNTs to improve electron conductivity. **(E)** A common strategy for applying nanomaterials in EC sensors: use GO in conjunction with 0D nanomaterials, which improves electrical conductivity, and modify the electrode. **(F)** A common strategy for applying nanomaterials in EC sensors: use NPs to modify carbon nanotubes and further improve their biocompatibility and conductivity. **(G)** A common strategy for applying nanomaterials in FL sensors: QDs combined with aptamers and functionalized that recognize specific antibiotics and fluoresce. **(H)** A common strategy for applying nanomaterials in FL sensors: as a detection platform, the 2D nanosheet has a fluorescence quenching effect. **(I)** A common strategy for applying nanomaterials in FL sensors: functionalized nanorods fluoresce in response to specific antibiotics.

The concept of nanomaterials began with nanocrystaline research by Gleiter and Marquardt (1984), Gleiter (1989), Gleiter (2000), and since then, nanomaterials have gradually become a hot topic of research. Nanomaterials can be divided into 0-dimensional (0D), 1-dimensional (1D), 2-dimensional (2D), and 3-dimensional (3D) in terms of dimensional characteristics. Dimensions refer to the number of dimensions where the material is not within the size boundary of 0.1–100 nm. Because their dimensions and structures are different, the sensitivity of sensors made of different nanomaterials is different according to the surface effect and the properties of the dielectric region (Figure 1).

The types of nanomaterials adopted for the determination of antibiotics are as follows. Among them, 0D materials include nanoclusters (Zang et al., 2013), nanoparticles (Wu et al., 2018), and quantum dots (QDs) (Malik et al., 2019); the 1D materials include nanowires (Shad et al., 2019), nanotubes (Xiao et al., 2007), and nanorods (Roushani and Ghanbari, 2018); the 2D materials include nanoflakes (Zeng et al., 2018) and nanofilms (Velusamy et al., 2018).

There are two common types of antibiotic detection sensors at present. One is an electrochemical (EC) sensor (Liu et al., 2019), and the other is a fluorescence (FL) sensor (Peng et al., 2018). The common characteristics of these two types of sensors are fast response, convenient operation, portability, and a low limit of detection (LOD). An EC sensor reflects the concentration of the measured object with electrical signals, so nanomaterials are commonly used to promote conductivity. Nanomaterials are often adopted in an FL as fluorescent materials or as fluorescence quenching materials, such as carbon quantum dots (CQDs) (Chen et al., 2018). The details of the types, detection limits, and measurement properties of nanomaterial sensors are given in Table 1.

**Table 1:** Characteristics of the different nanomaterials applied.

**Type**	**Nanomaterials**	**Role of nanomaterial**	**Analyte**	**References**
FL	0D CdSe–ZnS QDs	Sensitivity enhancement	Sulfamethazine	Ding et al., 2006
EC	1D MWCNTs	Sensitivity, selectivity enhancement	Oxytetracycline	Vega et al., 2007
EC	0D/1D AuNPs/SWCNTs	Sensitivity and stability enhancement	Chloramphenicol	Xiao et al., 2007
FL	0D CdSe–ZnS QDs	Sensitivity enhancement	Enrofloxacin	Chen et al., 2009
FL	0D CdTe QDs	Sensitivity enhancement	Etimicin	Wang et al., 2010
EC	2D GOs	Sensitivity and stability enhancement	Alpha fetoprotein	Wei et al., 2010
EC	0D/1D AuNCs/AuNRs	Selectivity enhancement	Ofloxacin	Zang et al., 2013
EC	1D/2D MWCNTs/GOs	Simplicity, stability enhancement	Azithromycin	Zhang et al., 2013
EC	2D CuO nanosheets	Sensitivity enhancement	Vancomycin	Khataee et al., 2014
EC	2D rGO nanosheet	Sensitivity enhancement	Rifampicin	Rastgar and Shahrokhian, 2014
EC	0D gold@silver nanoparticles	Sensitivity enhancement	Kanamycin	Zengin et al., 2014
FL	2D WS_2_ nanosheet	Selectivity and sensitivity enhancement	Bleomycin	Qin et al., 2015
EC	1D/2D MWCNTs/MoS_2_ nanosheets	Reproducibility and stability enhancement	Chloramphenicol	Govindasamy et al., 2017
EC	0D AuNPs	Sensitivities and specificity	Chloramphenicol	Huang et al., 2018
FL	0D CdTe QDs	Sensitivity enhancement	Sulfadiazine	Chen et al., 2018
EC	0D/2D g-C_3_N_4_ QDs/rGOs	Sensitivity enhancement	Sulfadimethoxine	Dang et al., 2018
EC	2D graphene	Stability and repeatability enhancement	Erythromycin	Huang et al., 2018
EC	0D Silver NPs	Speed and sensitivity enhancement	Ampicillin	Rosati et al., 2019
EC	0D CdS QDs	Selectivity and sensitivity enhancement	Chloramphenicol	Wang Y. et al., 2018
FL	0D g-C_3_N_4_ QDs	Sensitivity enhancement	Amikacin	Hassanzadeh et al., 2019
EC	0D/1D AuNPs/MWCNTs	Sensitivity enhancement	Oxytetracycline	He et al., 2019
EC	0D/2D AuNPs/GOs	Speed and sensitivity enhancement	Penicillin	Mohammad-Razdari et al., 2019b
EC	0D/2D AuNPs/rGOs	Reproducibility and selectivity enhancement	Sulfadimethoxine	Mohammad-Razdari et al., 2019a

*FL, fluorescence sensor; EC, electrochemical sensor; QDs, quantum dots; CNTs, carbon nanotubes; MWCNTs, multi-walled carbon nanotubes; SWCNTs, single-walled nanotubes; GOs, graphene oxides; AuNCs, gold nanoclusters; AuNPs, gold nanoparticles; AuNRs, gold nanorods; rGOs, redox graphene oxides*.

In the next section, we will introduce the application of 0D, 1D, and 2D nanomaterials in the two types of sensors, FL and EC. We will then summarize and forecast the status and trends of research in related fields in the third section.

## LD Nanomaterial-Based Sensors for Antibiotics Detection

### LD Nanomaterial-Based FL Sensors

FL sensors have high selectivity because the occurrence and quenching of fluorescence have specificity. Therefore, the use of FL sensors for the detection of antibiotics in complex media (such as milk, honey, etc.) has high research value, and it is one of the hot spots in the research field in recent years.

The most common 0D nanomaterials adopted in FL sensors are QDs (Liu et al., 2009). This is because of the long-term photostability, high-quantum yield, narrow emission, and broad excitation spectra of CdX QDs (Leptihn et al., 2010; Hou et al., 2011). QDs are generally composed of elements from groups III to V or II to IV in the periodic table of the elements (Zhang and Wei, 2016). QDs were applied as early as 2006. At that time, CdX (X = S, Se, etc.) QDs were mainly adopted (Qie Gen et al., 2007; Wang et al., 2009, 2010).

For example, Ding et al. adopted CdSe QDs and competitive fluorescence-linked immunosorbent assay (cFLISA) for the detection of sulfamethazine (Ding et al., 2006). QDs were applied as a fluorescence label in the cFLISA method. The combination of the cFLISA method and QDs has great specificity, so it can be used for the determination of antibiotic residues in chicken muscle. The LOD is as low as 3.6 × 10^−9^ M. In addition to QDs, other nanoparticles are also widely adopted in the design of antibiotic sensors. Berlina et al. integrated lateral flow assay with fluorescent labels, which provides the opportunity to achieve simple and sensitive control of milk contamination by chloramphenicol. This method also illustrates the high specificity of the combination of QDs and immunization methods. The LOD of chloramphenicol is 9.3 × 10^−9^ M (Berlina et al., 2013). NPs are also widely adopted in the design of antibiotic sensors. Mesoporous silica nanoparticles are an example of non-metal oxide nanoparticles that can be used (Wang C. et al., 2018). Wang et al. adopted aggregation-induced emission luminogens to functionalize silica nanoparticles. Because of the restricted intramolecular vibration and rotation in rigid silica networks, the material emitted strong blue fluorescence, which showed highly sensitive fluorescence-quenching responses toward nitrofuran antibiotics. The LOD of nitrofurazone reached 7.2 × 10^−6^ M.

1D nanomaterials have relatively few applications in the fluorescence detection of antibiotics. 1D nanomaterials like nanotubes or nanorods are more applied in EC sensors, which will be described in detail in the next section. Liu et al. developed a semiconductor of ZnO nanomaterials as a fluorescence sensor without leakage toxicity. ZnO nanomaterials have desirable optical and electronic properties, a large excitation binding energy of 60 m eV, and a wide bandgap. Liu adopted molecular imprinting polymer technology and ZnO nanomaterials to realize specific selectivity, high stability, and easy operation, which exhibited promising application in sensors (Liu et al., 2020).

Due to the superiority of 2D nanomaterials on the macro scale, they are easily adopted in sensing platforms. For example, Qin et al. made a fluorescent sensing platform for bleomycins by using WS_2_ nanosheets. In the presence of Fe(II), bleomycin forms a BLM·Fe(II) complex that reacts with oxygen to generate a BLM·Fe(III)OOH species. BLM·Fe(III)OOH can catalyze the incision of DNA. WS_2_ nanosheets exhibit different affinity toward single-stranded DNA (ssDNA) with different length and excellent fluorescence quenching ability. BLM catalyzes the incision of long ssDNA and restores fluorescence, Different fluorescence intensities correspond to different BLM concentrations. Using the above-described detection principle, the LOD of BLM is 3.0 × 10^−10^ M. Using rolling circle amplification to amplify the signal is an important factor in this method, and using ssDNA as a signal has good biological activity (Qin et al., 2015).

According to the literature summarized above, the nanomaterials used in FL are mainly 0D and 2D. 0D materials like QDs are rich in surface defects and are easy to modify and synthesize. They will emit high-intensity fluorescence when they encounter specific substances, which has good application value for antibiotics detection in complex environments. Compared with CdX QDs, CQDs are less biotoxic and have better biocompatibility. 2D materials are more suitable for building platforms, as they have good fluorescence quenching performance.

### LD Nanomaterial-Based EC Sensors

The characteristics of EC sensors are that they are fast, low-cost, high-sensitivity, and relatively portable, and they have hence been one of the development directions in environmental pollutant detection in recent years. In this part, we will mainly introduce the ways in which 0D, 1D, and 2D nanomaterials are used to improve the performance of EC sensors.

For 0D materials, QDs can be taken as an example. QDs can be used as stabilizers (Xu et al., 2018). Besides being adopted as stabilizers, Dang et al. adopted g-C_3_N_4_ QDs modified with redox graphene oxides (rGOs) in photoelectric sensors to measure sulfadimethoxine (SDM). g-C_3_N_4_ QDs possess good optical and electrical properties as compared to other carbon-based QDs (Dang et al., 2018). Due to their lower intrinsic conductivity, they need to be modified with materials with high conductivity and hydrophilic ability for better performance in the detection of antibiotics. By modifying g-C_3_N_4_ QDs with rGOs, a linear calibration range for SDM of 5.0 × 10^−10^ M−1.4 × 10^−15^ M was obtained, with a LOD of 1.0 × 10^−10^ M.

1D materials perform well in EC sensors. The general method of applying 1D nanomaterials in antibiotics sensing is through electrode surface modifications (Vega et al., 2007). Zhang et al. combined the hydrophilic properties of rGOs and the excellent electronic and antifouling properties of multi-walled carbon nanotubes (MWCNTs) for azithromycin detection. Compared with a bare glassy carbon electrode (GCE), the GCE modified with MWCNTs has a larger active surface area. The LOD of tetracyclines in a water sample was 1.1 × 10^−9^-6 × 10^−9^ M under pre-concentration (Zhang et al., 2013). P-type semiconductor-based transition metal oxide nanoparticles have received much attention due to their excellent physicochemical properties, which make them suitable for catalysis, energy storage, and electrochemical conversion applications. The integration of metal oxides and their synergistic effects enhance selectivity and activity. Chen et al. synthesized a CuO NPs@MWCNTs nanocomposite (Chen et al., 2019). AuNPs have also been introduced to anchor on MWCNTs, not only providing a large number of biological binding sites but also effectively improving the electron transfer rate (He et al., 2019).

In terms of cost or difficulty of preparation, carbon nanotubes are excellent choices, but nanorods and nanowires also have unique properties due to their morphologies. Nanowires can be easily synthesized to achieve a multisegment structure, which will enable the nanowire to be used for multifunctional applications, especially as high sensitivity, multitasking electrochemical biosensors (Li et al., 2019). Nanorods have a relatively small specific surface area but also have excellent electronic conductivity and are more stable.

0D, 1D, and 2D nanomaterials are used to improve the performance of electrochemical sensors. Herein, by viewing the reported studies, it can be seen that combinations of 0D (such as NPs) and 2D (rGOs) nanoparticles have attracted a lot of attention in this research field. Mohammad et al. fabricated an electrochemical aptamer-based biosensor on a pencil graphite electrode (PGE). The bare PGE was modified with rGOs and AuNPs for SDM determination. This optimizes the linear range of the electrode as well as stability and reproducibility (Mohammad-Razdari et al., 2019b).

## Conclusion and Outlook

### Supplementary Notes on Methods of Applying LD Nanomaterials in Antibiotic Detection

For 0D materials, QDs are often used as fluorophores with excellent performance, and metal or metal oxide nanoparticles are often used to modify other materials to further improve electronic conductivity. For 1D materials, nanotubes, nanowires, and nanorods are often used to enhance sensor performance, or to form microelectrode arrays; 2D materials, meanwhile, are more convenient for constructing platforms (Wang et al., 2017; He and Yan, 2018). Sensitivity is usually enhanced by means of signal amplification and enhancement (Wei et al., 2010). In order to improve the performance of the sensor, combination of nanomaterials in various dimensions is a very common method, such as using AuNPs to modify rGOs to improve the electronic conductivity of the sensor, which promotes the stability and sensitivity of the sensor. Good electron transfer capabilities and conductivity can also promote selectivity (Munawar et al., 2018).

When designing sensors, fluorescence immunoassay (FIA) is one of the promising technologies for antibiotics detection based on LD nanomaterials. In order to cope with complex environments, the sensors need to have good selectivity (Govindasamy et al., 2017). For example, FIA, a type of immunoassay, has two models: competitive type and sandwich type. The competitive type refers to a competitive mechanism that enables unlabeled antigens to compete with labeled antigens by binding to a limited antibody. For the competitive type, unlabeled antigens, and labeled antigens compete with antibodies that are bound to QDs. The fluorescence intensity varies with different antigen concentrations (Chen et al., 2009; Zhu et al., 2011).

For the sandwich type, the reaction principle is illustrated as follows. An excess of antibody is fixed on the immune reaction carrier, and subsequently, a certain amount of antigen is added. After the immune reaction, an excess of labeled antibody is added to form a “sandwich” immune complex. The more antigens in the sample and the more labeled antibodies bound, the stronger the labeled fluorescent signal of the sandwich immune complex is. Zengin et al. adopted this method and made a sensor on the basis of gold and silver nanoparticles. Accordingly, the LOD of kanamycin can reach 3.4 × 10^−12^ M (Zengin et al., 2014).

This method can be adopted in high-precision detection of antibiotics in complex media; such applications have been introduced above in the 0D section.

### Summary and Outlook

Due to their different morphologies and dimensions, different nanomaterials have different beneficial properties.

The development of antibiotics sensors in the next few years can be roughly divided into the following directions: the pursuit of high-sensitivity detection, the pursuit of low-cost and fast on-site detection, and the pursuit of multi-functional integration. Among them, the gradual realization of low-cost and rapid monitoring should be the most noteworthy, and this fits with the advantages of EC. According to the 57 articles cited in this article, we concluded that, in FL, 0D is currently used most, followed by 2D, and 1D is used least. 0D is represented by various types of QDs that can be used as fluorophores. In EC, 1D is the most used type, and 2D and 0D are used relatively little. In the future, researchers who want to improve FL performance can pay more attention to new quantum dots and NPs, and researchers who want to improve EC performance can pay more attention to materials with good electron conduction properties such as CNTs. At the same time, the performance improvement brought about by NP modification of other nanomaterials should also be considered. Finally, when the wish is to build a platform, 2D materials have advantages in terms of morphology. At present, there are few related studies, and in the coming decades, these may be hot research topics (Rastgar and Shahrokhian, 2014; Kokulnathan and Chen, 2020; Liu et al., 2020; Roushani et al., 2020).

In order to further optimize the performance of antibiotics sensors based on LD nanomaterials, the following suggestions are proposed according to the application status:

1. Fluorescence immunoassay method can be better applied to the detection of low levels of antibiotics in complex media, such as chicken muscle, milk, and honey. This is because quantum dots can be well combined with antibody immunoassay. This method has great specificity, lower LOD, and good biocompatibility. In order to achieve specific functions for various antibiotics, different immunoassays combined with QDs need to be designed.
2. Pay attention to the portability, fastness of measurement, and cooperation with smart devices (Tian et al., 2015). Taking the research by Yu et al. As an example, the electrodes were printed by screen printing technology to achieve rapid response measurement within 30 s (Yu et al., 2019). The research of Wang et al. in 2020 realized online monitoring through the color selection application of the mobile phone and the fluorescence reaction. These studies have good application value.
3. Build a platform that can detect multiple antibiotics at the same time. The platformization of the sensor depends on 2D nanomaterials or MOF. MOF is often used in combination with metal nanoparticles to obtain better performance. To achieve the simultaneous measurement of multiple antibiotics, microarray electrode technology can achieve this complex function.
4. In addition to the combination with MOF, the combination of NPs and other materials can also achieve better performance, which is a breakthrough direction for high-precision research.

## Author Contributions

YW and FL supervised this work. YW established the conceptualization of this work and acquired the funding. YD did research on methodology, collected the data, edited, and wrote this review. All of the authors read and approved the manuscript.

## Conflict of Interest

The authors declare that the research was conducted in the absence of any commercial or financial relationships that could be construed as a potential conflict of interest.
